# Altered functional and structural brain network organization in autism^☆^

**DOI:** 10.1016/j.nicl.2012.11.006

**Published:** 2012-11-16

**Authors:** J.D. Rudie, J.A. Brown, D. Beck-Pancer, L.M. Hernandez, E.L. Dennis, P.M. Thompson, S.Y. Bookheimer, M. Dapretto

**Affiliations:** a Brain Mapping Center, UCLA, Los Angeles, CA, USA; b Interdepartmental Neuroscience Program, UCLA, Los Angeles, CA, USA; c David Geffen School of Medicine, UCLA, Los Angeles, CA, USA; d Center for Cognitive Neuroscience, UCLA, Los Angeles, CA, USA; e Laboratory of Neuro Imaging, Department of Neurology, UCLA, Los Angeles, CA, USA; f Department of Psychiatry & Biobehavioral Sciences, Semel Institute for Neuroscience and Human Behavior, UCLA, Los Angeles, CA, USA

**Keywords:** Resting-state functional connectivity, Diffusion tensor imaging, Graph theory, Brain networks, Autism spectrum disorders

## Abstract

Structural and functional underconnectivity have been reported for multiple brain regions, functional systems, and white matter tracts in individuals with autism spectrum disorders (ASD). Although recent developments in complex network analysis have established that the brain is a modular network exhibiting small-world properties, network level organization has not been carefully examined in ASD. Here we used resting-state functional MRI (n = 42 ASD, n = 37 typically developing; TD) to show that children and adolescents with ASD display reduced short and long-range connectivity within functional systems (i.e., reduced functional integration) and stronger connectivity between functional systems (i.e., reduced functional segregation), particularly in default and higher-order visual regions. Using graph theoretical methods, we show that pairwise group differences in functional connectivity are reflected in network level reductions in modularity and clustering (local efficiency), but shorter characteristic path lengths (higher global efficiency). Structural networks, generated from diffusion tensor MRI derived fiber tracts (n = 51 ASD, n = 43 TD), displayed lower levels of white matter integrity yet higher numbers of fibers. TD and ASD individuals exhibited similar levels of correlation between raw measures of structural and functional connectivity (n = 35 ASD, n = 35 TD). However, a principal component analysis combining structural and functional network properties revealed that the balance of local and global efficiency between structural and functional networks was reduced in ASD, positively correlated with age, and inversely correlated with ASD symptom severity. Overall, our findings suggest that modeling the brain as a complex network will be highly informative in unraveling the biological basis of ASD and other neuropsychiatric disorders.

## Highlights

► Complex network analysis of resting-state fMRI and DTI tractography in autism ► Local and long-range functional connectivity is reduced in ASD. ► Reduced local efficiency and modularity of functional networks in ASD ► Altered age-related trajectory of global efficiency for structural networks in ASD

## 1 Introduction

Autism spectrum disorders (ASD) are increasingly prevalent neurodevelopmental disorders (Kim et al., 2011) characterized by atypical social behavior, including deficits in receptive and expressive language, theory of mind, and mental flexibility. Findings of functional underconnectivity between brain regions in individuals with ASD relative to matched controls have been reported as they perform a variety of cognitive tasks (see Schipul et al., 2011, for review). Multiple studies have found that task-independent (i.e., intrinsic) functional connectivity, including interhemispheric (Anderson et al., 2011a) and default mode network (DMN) connectivity is also lower in ASD (e.g., Kennedy and Courchesne, 2008). Further supporting an underconnectivity theory, diffusion tensor imaging (DTI) studies have found reductions in structural white matter integrity across most major tracts (see Vissers et al., 2012, for review).

In addition to reports of reduced functional connectivity *within* major networks (i.e., functional integration), connectivity *between* different networks (i.e. functional segregation) is altered in ASD (Rudie et al., 2012a). Functional brain networks become simultaneously more integrated and segregated during typical development (e.g., Fair et al., 2009) and white matter integrity increases during development (e.g., Lebel et al., 2012), suggesting that brain networks in ASD may reflect ‘immature’ or aberrant developmental processes.

Despite this array of regional and systems level findings in ASD, it is unclear how these alterations might be reflected at a network level where the brain is modeled as a network of hundreds of interacting regions composing several integrated and segregated systems. Graph theory, which describes complex systems as a set of “nodes” (i.e., brain regions) and “edges” (i.e., connections between nodes), has characterized the brain as a complex network with a hierarchical modular organization consisting of several major functional communities (i.e., visual, sensorimotor, default mode, and attentional systems; see Wang et al., 2010, for review). Structural and functional brain networks exhibit robust levels of local and global efficiency (i.e., small-world properties; Watts and Strogatz, 1998) that can be quantitatively characterized using graph theoretical methods (Bullmore and Sporns, 2009; Rubinov and Sporns, 2009). Structural and functional graph theoretical studies have begun to map how local and global network properties change during development (Fair et al., 2009; Hagmann et al., 2010), aging (e.g., Meunier et al., 2009) and in diseases such as schizophrenia (e.g., Bassett et al., 2008) and Alzheimer's (e.g., Supekar et al., 2008).

In this study we sought to compare functional and structural connectivity in children and adolescents with ASD relative to typically developing (TD) children by characterizing local and global graph theoretical metrics of structural and functional networks using a recently validated 264-region functional parcellation scheme (Power et al., 2011). We first compared simpler network connections and then characterized higher-level network properties including clustering, characteristic path length, small worldness and modularity. Additionally, since structural connectivity has been shown to correlate with functional connectivity (Hagmann et al., 2008; Honey et al., 2009), we wanted to determine whether structure–function correlations differed between groups and how functional and structural network properties relate to each other across development in TD and ASD individuals.

## 2 Materials and methods

### 2.1 Subjects

High-functioning children and adolescents with ASD, as well as TD children and adolescents, were recruited through UCLA's Center for Autism Research and Treatment (CART) and flyers posted throughout the greater Los Angeles area. Individuals with metal implants, psychiatric or neurologic disorders, structural brain abnormalities, or known genetic conditions were excluded from participation. Informed consent and assent to participate was obtained prior to assessment according to protocols approved by the UCLA Institutional Review Board (IRB). Verbal, performance, and overall intelligence were assessed for each participant using the Wechsler Abbreviated Scale of Intelligence (WASI; Wechsler, 1991) or the full Wechsler Intelligence Scale for Children (WISC; Wechsler, 1999). High-functioning children with ASD had a prior clinical diagnosis of autism based on criteria from the Diagnostic and Statistical Manual of Mental Disorders (DSM IV), which was confirmed with the Autism Diagnostic Observation Scale (ADOS-G; Lord et al., 2000) and/or Autism Diagnostic Interview (ADI-R; Lord et al., 1994).

A total of 60 individuals with ASD (52 males and 8 females) and 45 TD individuals (38 males and 7 females) were included in either the resting state, DTI or combined resting state/DTI final matched datasets (Table 1). After excluding subjects with excessive head motion, the resting state sample included 42 ASD subjects and 37 TD subjects and the DTI sample included 51 ASD subjects and 43 TD. Both structural and functional data were available for 35 ASD and 35 TD subjects. The three sets of matched groups did not significantly differ based on age, sex, mean/maximum head motion, or full-scale, verbal and performance IQ (Table 1).

Twenty-two individuals with ASD and one TD individual reported the use of one or more psychotropic medications. One TD subject was using a psychostimulant. Of the subjects in our ASD sample, 12 were taking psychostimulants, 5 were taking sympatholytics, 9 were taking atypical antipsychotics, 9 were taking selective serotonin reuptake inhibitors, 3 were taking selective norepinephrine reuptake inhibitors, 3 were taking an atypical antidepressant, and 2 were taking anticonvulsants. There were no significant differences (*p*s > 0.30) between medicated and unmedicated ASD individuals for each of the functional and structural measures described in the following sections.

### 2.2 MRI data acquisition

All resting-state fMRI and DTI scans were acquired on a Siemens 3 T Trio at UCLA. A scout localizing scan was collected to help prescribe the orientation of the scans. Next, a matched bandwidth T2-weighted high-resolution echo planar scan was acquired co-planar to the functional images, which ensures identical distortion characteristics for registration purposes (Siemens 3 T Trio: TR = 5000 ms, TE = 34 ms, matrix size: 128 × 128, 19.2 cm FoV, and 36 4-mm thick slices with an in-plane voxel dimension of 1.50 × 1.50 mm). In a single session, subjects were asked to relax and keep their eyes open while a fixation cross was displayed on a white background for 6 min (T2*-weighted functional images: TR = 3000 ms, TE = 28 ms, matrix size 64 × 64, 19.2 cm FoV, and 34 4-mm thick slices (no gap), interleaved acquisition, with an in-plane voxel dimension of 3.0 × 3.0 mm). The DTI sequence consisted of 32 scans with different diffusion-weighted directions (*b* = 1000 s/mm^2^), three scans with no diffusion sensitization, at *b* = 0, and additional six scans at *b* = 50 s/mm^2^. Other parameters were TR = 9500 ms, TE = 87 ms, GRAPPA on, FOV = 256 mm, with 75 slices, yielding an in-plane voxel dimension of 2 × 2mm with 2-mm thick axial slices, and total scan time = 8 min 1 s.

### 2.3 Resting state fMRI preprocessing

Imaging data were analyzed using FSL version 4.1.4 (FMRIB's Software Library, www.fmrib.ox.ac.uk/fsl; Smith et al., 2004) and AFNI (Analysis of Functional NeuroImages; Cox, 1996). Structural and functional images were skull-stripped using AFNI (3dskullstrip and 3dautomask). Functional volumes were motion corrected to the mean functional volume with MCFLIRT (Motion Correction using FMRIB's Linear Image Registration Tool) using a normalized correlation ratio cost function and sinc interpolation (Jenkinson et al., 2002). Translations and rotations in the *x*, *y*, and *z* dimensions were calculated from volume to volume and averaged to generate mean and max relative displacement values, which did not significantly differ between the final matched groups (Table 1). Subjects with a single displacement (combined translational and rotational movements) greater than 2.5 mm (13 ASD and 5 TD) were excluded prior to further analyses and not included in the final samples. Images were spatially smoothed using a Gaussian kernel of FWHM 5 mm. A band pass filter (0.1 Hz > *t* > 0.01 Hz) was applied to the data in order to minimize the effects of cardiac and respiratory fluctuations. The 6 rigid body motion parameters and average white matter (WM), cerebrospinal fluid (CSF), and global time-series and their temporal derivatives were then regressed out of the data. The WM and CSF time-series reflected signal from subject-specific regions of interest created using FAST (FSL's Automatic Segmentation Tool). Given the recent concerns regarding the effect of motion in resting state fMRI connectivity (Power et al., 2012; Van Dijk et al., 2012), in addition to matching the groups by mean and maximum relative head motion, we also regressed out individual volumes with large signal intensity changes (i.e., motion spikes) by creating additional nuisance regressors that modeled individual time points with greater than half of a standard deviation change in global signal intensity.

### 2.4 Resting state fMRI connectivity matrix construction

One major methodological hurdle in graph theory approaches to neuroimaging concerns how to define the nodes of the network (Wang et al., 2009; Zalesky et al., 2010; Craddock et al., 2011; Power et al., 2011). Most studies have used anatomical atlases (e.g., He et al., 2007) or individual voxels (e.g., van den Heuvel et al., 2008) as nodes. However, anatomical atlases include relatively large regions that are likely to contain multiple functional regions, which can distort/obscure true properties of the network by mixing distinct signals (Butts, 2009; Smith et al., 2010; Craddock et al., 2011; Power et al., 2011). Conversely, voxel-wise parcellation approaches can be biased by artificially strong local connections (Power et al., 2011, 2012). A whole-brain parcellation scheme was recently created based on a large meta-analysis of fMRI studies combined with whole brain functional connectivity mapping (Power et al., 2011). This set of 264 putative functional regions was shown to more accurately represent the information present in the network (i.e., it was better at detecting previously characterized functional networks such as dorsal and ventral attention subnetworks) relative to voxelwise and atlas-based parcellation approaches. Therefore, we chose this set of 264 regions for whole-brain parcellation. For each subject, 5-mm radius spheres based on the MNI coordinates of these 264 regions (Power et al., 2011) were registered to functional space (12 DOF, affine, and correlation ratio cost function) through registration from the MNI 152 template to the high-resolution echo-planar (12 DOF, affine, and mutual information cost function) using FSL's Linear Image Registration Tool (FLIRT). We then correlated timeseries between each of the 264 brain regions and z-transformed correlation coefficients in order to generate 264 × 264 whole brain functional connectivity matrices for each subject. Graph theoretical metrics and statistics were computed with Matlab (The Mathworks, Natick, MA) using the Brain Connectivity Toolbox (Rubinov and Sporns, 2009).

**Fig. 1:**
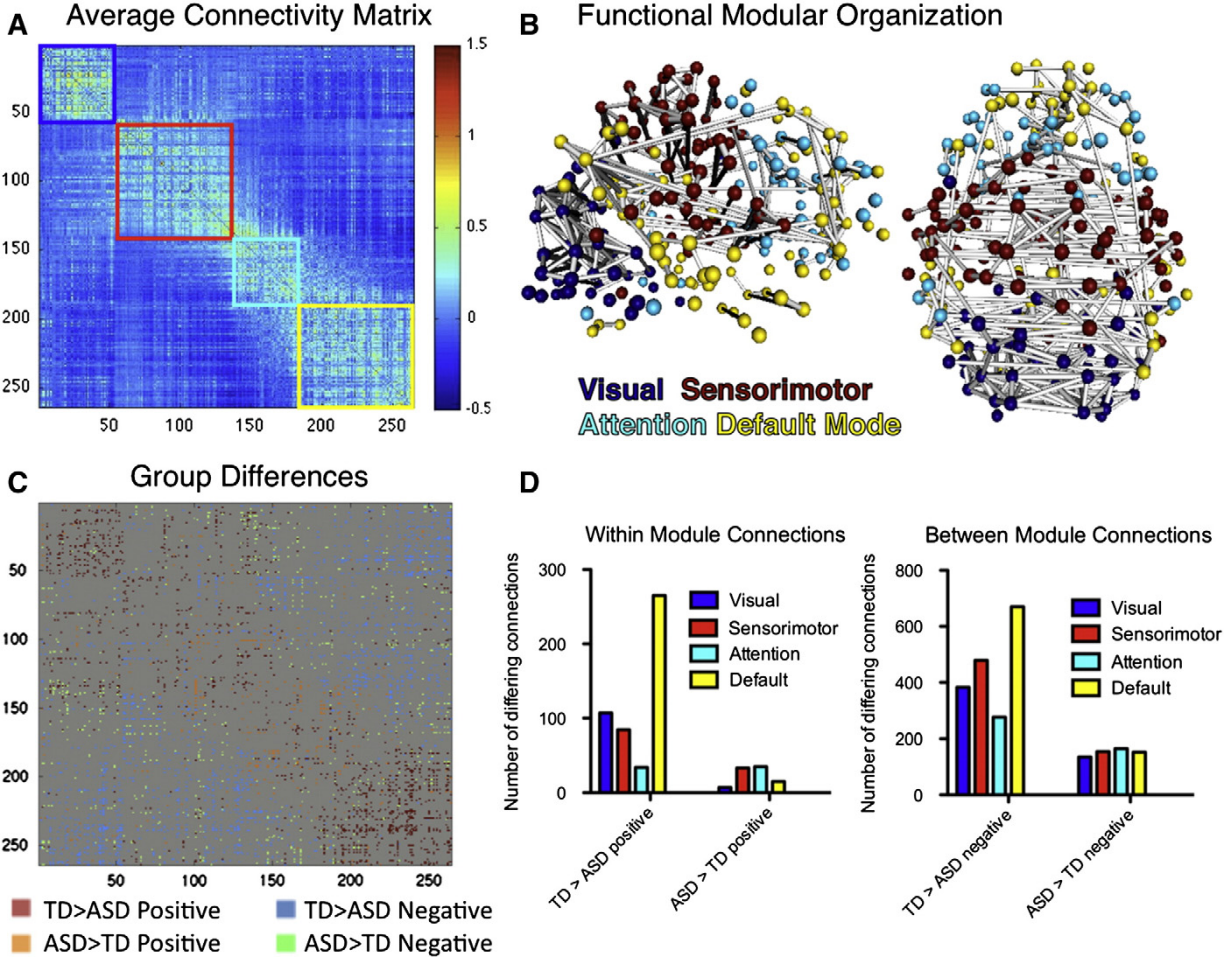
Functional network organization. (A) Average functional connectivity matrix reorganized by its modular organization with colored boxes around each of the four communities (visual = blue, sensorimotor = red, attention/control = cyan, and default = yellow). (B) Three dimensional sagittal and axial views of the functional graph in anatomical space displaying top 2% of connections and nodes colored by community. (C) Functional connectivity matrix group differences (*p* < 0.05 uncorrected) displaying typically developing (TD) > Autism Spectrum Disorder (ASD) for positive (red), ASD > TD for positive (orange), TD > ASD for negative (blue) and ASD > TD for negative (green). (D) Numbers of TD > ASD and ASD > TD between group connections differing for within group positive connections (left) and between group negative connections (right).

Before comparing graph theoretical properties, we sought to investigate pairwise differences in connection strengths as a function of the network's modularity, which refers to the set of subnetworks or distinct communities that exist within the network as a whole. Constructing the most representative modularity partition is an active area of research (http://arxiv.org/abs/1206.4358); however, at present there is no single, agreed-upon method for choosing the most representative partition. Here we used the Louvain modularity algorithm (Blondel et al., 2008) applied to the unthresholded functional connectivity matrix averaged across all subjects after removing all negative weights. Although there is some recent work regarding how to incorporate negative weights into graph theoretical metrics (Rubinov and Sporns, 2011; Schwarz and McGonigle, 2011), we chose to use well-established algorithms that use only positive connections in the calculation of graph theoretical metrics. We chose a random partition containing 4 modules (the most common number of modules identified over 100 runs of the algorithm) as our representative partition and reorganized the order of nodes in the functional connectivity matrix by this modular organization for visualization purposes (Fig. 1A). This representative partition was also used to determine whether each connection was a within- or between-module connection for additional calculations described in the next paragraph. The similarity of this chosen modularity partition with 99 other modularity iterations of the group average matrix was calculated using normalized mutual information (NMI; Meilă, 2007). Additionally, given the controversy regarding the accuracy of comparing modularity of group average matrices (Simpson et al., 2012) with individual subject matrices, we compared the similarity of each individual's modular organization with that of our representative group average modular organization with NMI (Meilă, 2007).

**Fig. 2:**
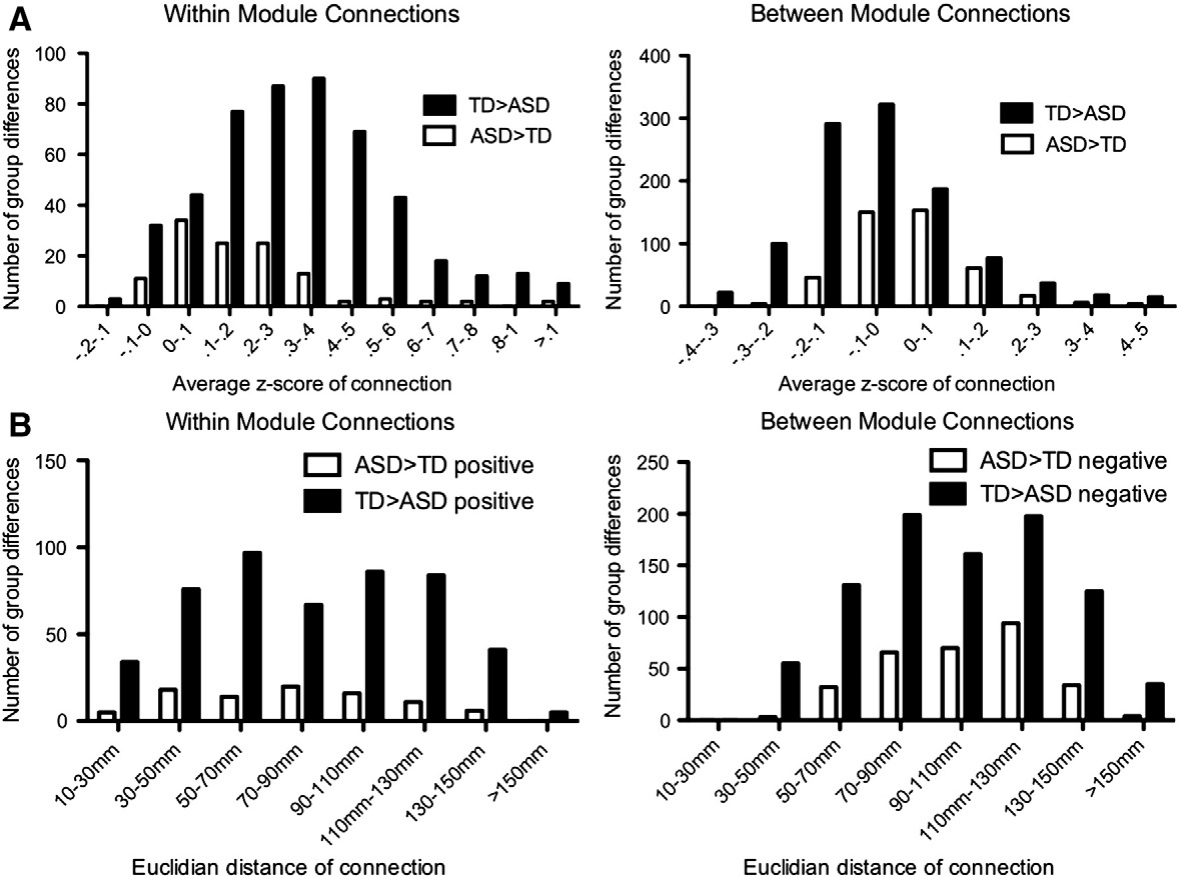
Distribution of functional connectivity differences. (A) Numbers of connections with significant group differences for typically developing (TD) > Autism Spectrum Disorder (ASD; black) and ASD > TD (white) displayed as a function of average connectivity strength across all subjects and (B) average Euclidean distance for within-module connections (left) and between-module connections (right).

In order to interrogate pairwise differences in connection strengths between the two groups, two-sample *t*-tests were performed for every z-transformed connection strength value (Fig. 1C). The significance was set at *p* < 0.05, uncorrected, for these initial exploratory analyses. If there was significantly lower connectivity in a connection for the ASD or TD group (vs. the other group) that had an average correlation value below zero, it was categorized as stronger negative connectivity for that group (as opposed to stronger positive connectivity for the other group, which is mathematically equivalent) given that negative or anticorrelations are likely to represent real phenomenon (Chang and Glover, 2009; Anderson et al., 2011c; Smith et al., 2012) and stronger connectivity for one group simply represent weaker negative connectivity for the other group (Anderson et al., 2011b; Rudie et al., 2012a). The number of connections differing between groups was assessed for each of the identified modules both for within-module positive connections and between-module negative connections (Fig. 1D). Numbers of within-module positive and between-module negative connections differing between groups were compared and displayed as a function of the connection's average *z*-transformed correlation value (Fig. 2A) and Euclidean distance between regions (Fig. 2B).

### 2.5 Resting state fMRI connectivity graph theoretical analyses

**Fig. 3:**
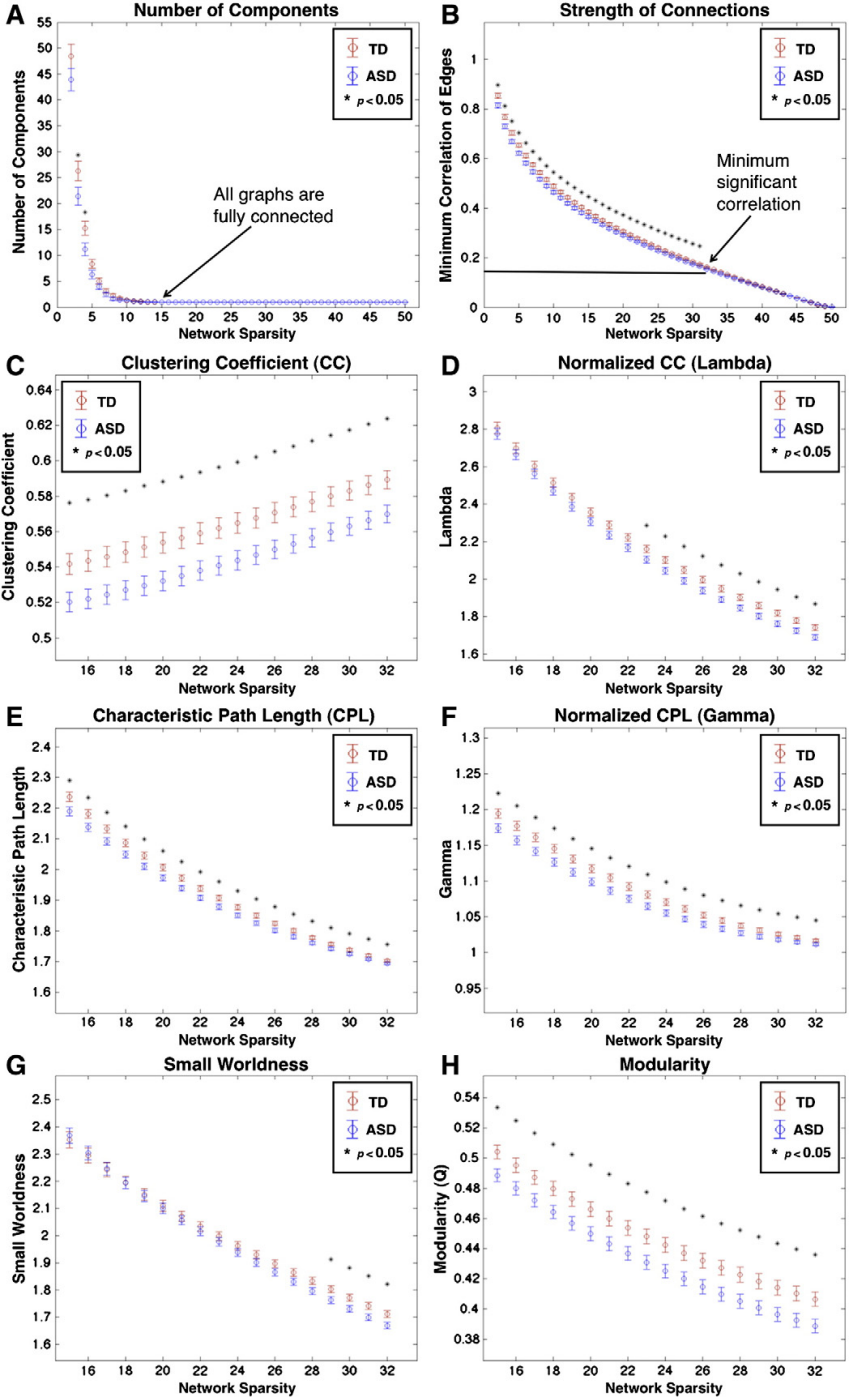
Graph theoretical metrics of functional networks. (A) Average and standard error for TD (red) and ASD (blue) number of components, (B) minimum correlation coefficient for edges, (C) clustering coefficient, (D) gamma, (E) characteristic path length, (F) lambda, (G) small worldness and (H) modularity Q values as a function of network sparsity. Number of components and minimum correlation strength are shown between 1% and 50% network sparsity in 1% increments while other network properties are displayed between 15% and 32% network sparsity in 1% increments (equivalent to minimum correlation values of 0.34 and 0.15). Significant between group differences (*p* < 0.05) are indicated by *.

As there is no rationale for using a particular cutoff for functional connectivity strength to determine whether an edge exists in a functional network, we compared local and global network properties over a range of functional connection thresholds. Thresholding a network based on correlation strength can yield different network sparsities (number of existing edges divided by number of possible edges), which influence network properties and can bias a comparison of graph metrics between groups (Ginestet et al., 2011; Schwarz and McGonigle, 2011; Bassett et al., 2012). In fact, we found that at higher z-correlation thresholds the TD group had a higher average sparsity (Fig. 3A). Therefore, we chose to equalize network sparsity between subjects by taking an equivalent percentage of the strongest positive connections (negative connections were ignored) for each subject and binarizing the network weights before calculating graph theoretical metrics. Binarization is a common step in functional graphs (e.g., Achad and Bullmore, 2007; Supekar et al., 2008) in order to preserve only the strongest (most probable) functional connections and treat these connections equivalently. We examined functional network properties between 15% and 32% sparsity. The upper threshold of 32% was chosen because the weakest edge it includes corresponds to a correlation coefficient of 0.15, which is the minimum correlation needed to be statistically significant (*p* < 0.05) across 120 functional images. At lower sparsity levels, network properties begin to break down as the network becomes fragmented. Therefore, we chose 15% sparsity (corresponding to a minimum correlation coefficient of 0.34 (*p* < .001)) as the low end of the range based on the requirement that all individual subject graphs be fully connected (Fig. 3B).

**Fig. 4:**
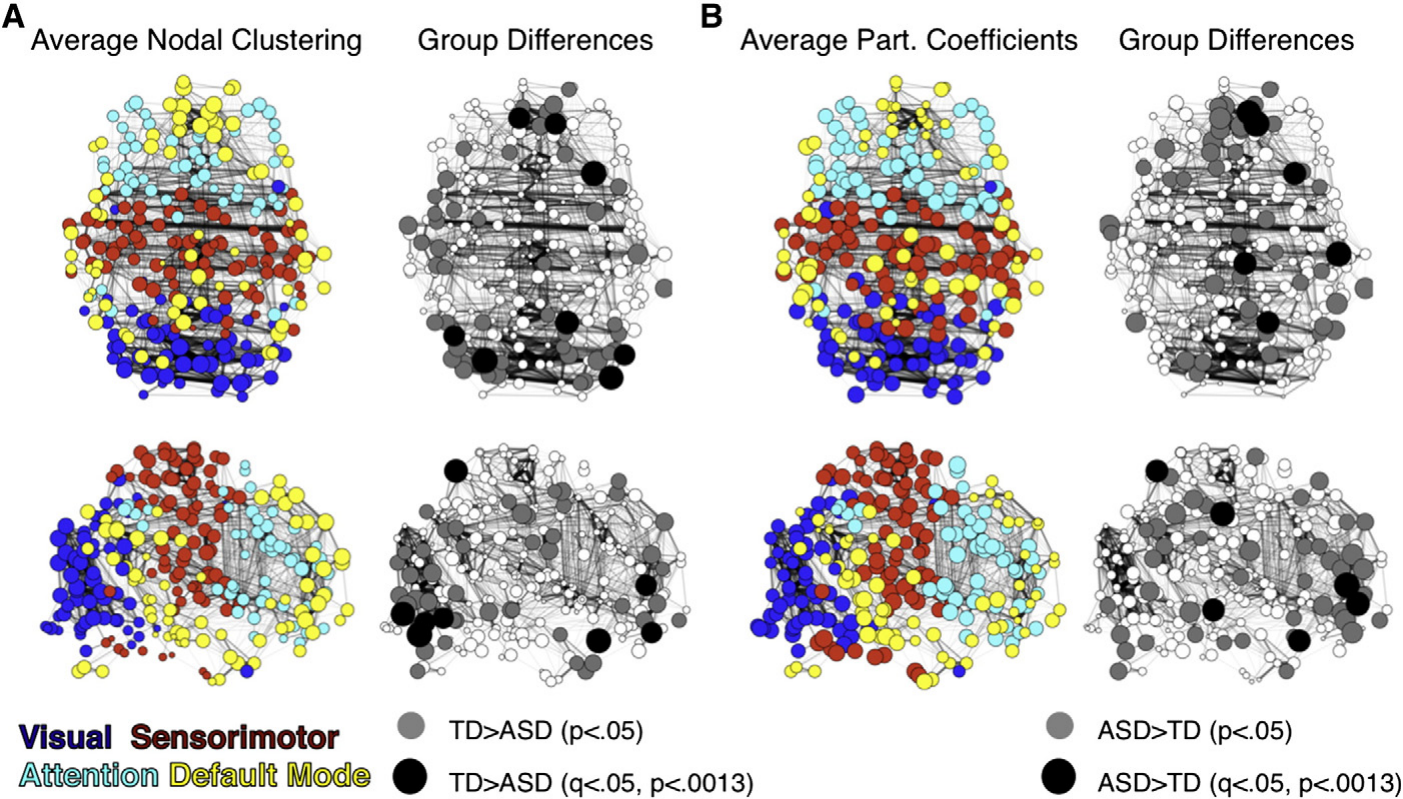
Nodal differences in clustering and participation coefficients. (A) Two dimensional axial and sagittal views of the functional graph in anatomical space displaying top 5% of connections with nodes colored by community organization (left columns) and radii proportional to average and significant between group differences (*p* < 0.05 in gray, FDR corrected *q* < 0.05 (*p* < 0.0013) in black; right column) for nodal clustering (TD > ASD) and (B) participation coefficients (ASD > TD).

We focused on 6 global graph theoretical metrics (see Rubinov and Sporns, 2009 for formulas of these metrics). These metrics were: *clustering coefficient* (CC), which measures how much neighbors of a node are connected to each other and is closely related to local efficiency; *characteristic path length* (CPL), which is the average number of edges needed to get from any node in the network to any other node in the network and is inversely related to global efficiency; normalized CC and CPL (*lambda* and *gamma*), which are calculated as the ratios of CC or CPL to the average CC or CPL from simulated random networks; *small worldness*, which is the ratio of *lambda* to *gamma* (Humphries et al., 2006); and *modularity* Q values, which represent the proportion of within-module edges in the network minus within-module edges calculated from a similar random network (Newman, 2006). For the calculation of *lambda* and *gamma*, we randomized networks by starting with a true network and then performing random double edge swaps with the constraint that these swaps must maintain the connectedness of the network. This algorithm preserved the degree of each node in the true network and was performed with the randmio_und_connected.m script in the Brain Connectivity Toolbox. One hundred of these random networks were calculated for each subject. *Lambda* and *gamma* were calculated using the mean of the C and L from the random networks. Since modularity Q values can vary based on random differences in module assignments from run to run, Q values were averaged over 100 iterations of the algorithm. All metrics were averaged across 15% to 32% sparsities in 1% increments to generate average values for each metric given the smooth curve across the sparsity range (Fig. 3). Two sample *t*-tests were performed on these metrics between subjects at each sparsity level (Fig. 3C–H) and for metrics averaged across sparsity levels (Table 2). To correct for multiple comparisons across the 6 metrics, False Discovery Rate (FDR *q* < 0.05; Benjamini and Hochberg, 1995; Storey and Tibshirani, 2003) was applied. For each node, clustering coefficients, participation coefficients and betweenness centrality were also averaged across sparsity levels for each subject and compared between groups (Fig. 4A). Betweenness centrality measures how often the shortest path goes through a given node while participation coefficients reflect how much a node interacts with nodes in different communities (Guimerà et al., 2005) and each roughly corresponds to global metrics of characteristic path length and modularity, respectively. Differences in nodal metrics are shown at more stringent (FDR: *q* < 0.05) and less stringent thresholds (*p* < 0.05, uncorrected).

### 2.6 Diffusion MRI preprocessing

Individual volumes with gross motion artifacts were excluded from further analysis and subjects with excessive motion (greater than 8 volumes (20%) with motion artifacts) were not included in final samples (6 ASD and 3 TD). Motion and eddy current correction was performed on the diffusion-weighted images using eddy_correct in FMRIB's Diffusion Toolbox (FDT), while MCFLIRT was used to quantify mean and maximum relative motion (Table 1), which did not differ between groups. Dtifit was used to fit a diffusion tensor model to the data at each voxel and calculate voxelwise Fractional Anisotropy (FA) values for each subject. Whole brain deterministic tractography was then performed using the fiber assignment by continuous tracking (FACT) algorithm (Mori and van Zijl, 2002) in Diffusion Toolkit (http://trackvis.org/dtk). We sought to boost the likelihood of detecting longer fibers between spatially separate spherical ROIs by relaxing constraints on our tractography algorithm. Therefore, tractography was carried out by propagating fibers from each voxel with a maximum turn angle of 50° (Zalesky et al., 2010; Brown et al., 2011) and without an FA cutoff. The spatial separation of the ROIs effectively acts as a filter and offsets the reduced constraints placed on tractography as, with greater distance, it becomes less likely that spurious fibers will continue to propagate and connect distant ROIs. Fibers were smoothed using a spline filter. Fibers shorter than 5 mm were excluded as this corresponds to 2 voxels, for which a turn angle cannot be determined.

### 2.7 Diffusion MRI fiber connectivity matrix construction

We used the same set of 264 coordinates from Power et al. (2011) to generate 10 mm radius spheres in MNI space. Dilating spheres to 10 mm radii (relative to 5 mm radii spheres for functional nodes) ensured inclusion of nearby white matter fibers given that nodal coordinates were centered in gray matter. This set of nodes covers 50.6% of all white matter voxels based on FSL's white matter tissue priors thresholded at 50%. Additionally, on average 60.9% of the voxels in each ROI were white matter voxels. These 264 masks were transformed to each subject's diffusion space (12 DOF, affine, and correlation ratio cost function) through registration to the hires image (12 DOF, affine, and mutual information cost function). In order to generate edges between nodes of structural networks, the number of fibers connecting each region was counted. A fiber was defined as connecting two regions if one fiber endpoint terminated within one region and the other endpoint terminated within the other region. This process was repeated using all 264 regions as seeds in order to derive a 264 × 264 whole brain structural connectivity matrix for each subject, using custom software written for this purpose (UCLA Multimodal Connectivity Package; http://github.com/jbrown81/umcp). Additionally, average FA and mean diffusivity (MD) were calculated for each connection.

**Fig. 5:**
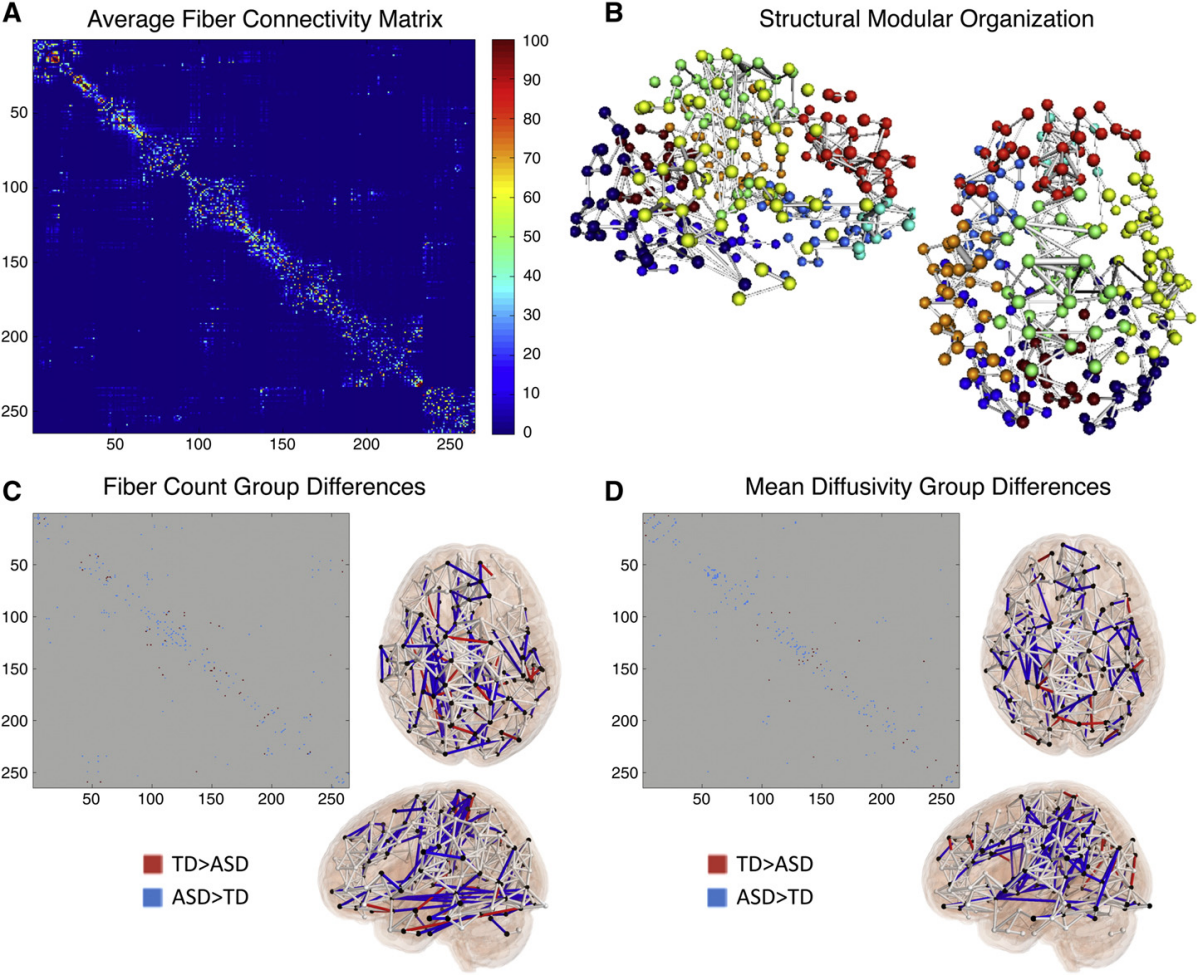
Structural network organization. (A) Average structural connectivity matrix reorganized by its modular organization. (B) Three dimensional sagittal and axial views of the structural network in anatomical space displaying top 2% of connections. (C) Structural connectivity matrix group differences (*p* < 0.05, uncorrected) displaying typically developing (TD) > Autism Spectrum Disorder (ASD) for fiber counts and (D) mean diffusivity in the connectivity matrix and in 3D brain space.

The Louvain modularity algorithm (Blondel et al., 2008) was run on the group average unthresholded fiber connectivity matrix. The order of nodes in the fiber connectivity matrix was reorganized based on a representative modularity partition with 9 modules (Fig. 5A). The similarity of this representative average modularity partition was compared with each individual matrix's modularity partition as well as 99 additional runs of the modularity algorithm on the average matrix using normalized mutual information (Meilă, 2007).

Two sample *t*-tests (*p* < 0.05, uncorrected for initial exploratory analyses) were performed on fiber counts, FA, and MD for every connection after masking by connections that have an average of 5 or more fibers (5.75% of all possible connections; Fig. 5B,C) in order to minimize false positive connections. Connections differing between groups for number of fibers and MD were compared as a function of average fiber count and Euclidean distance.

### 2.8 Structural connectivity graph theoretical analyses

**Fig. 6:**
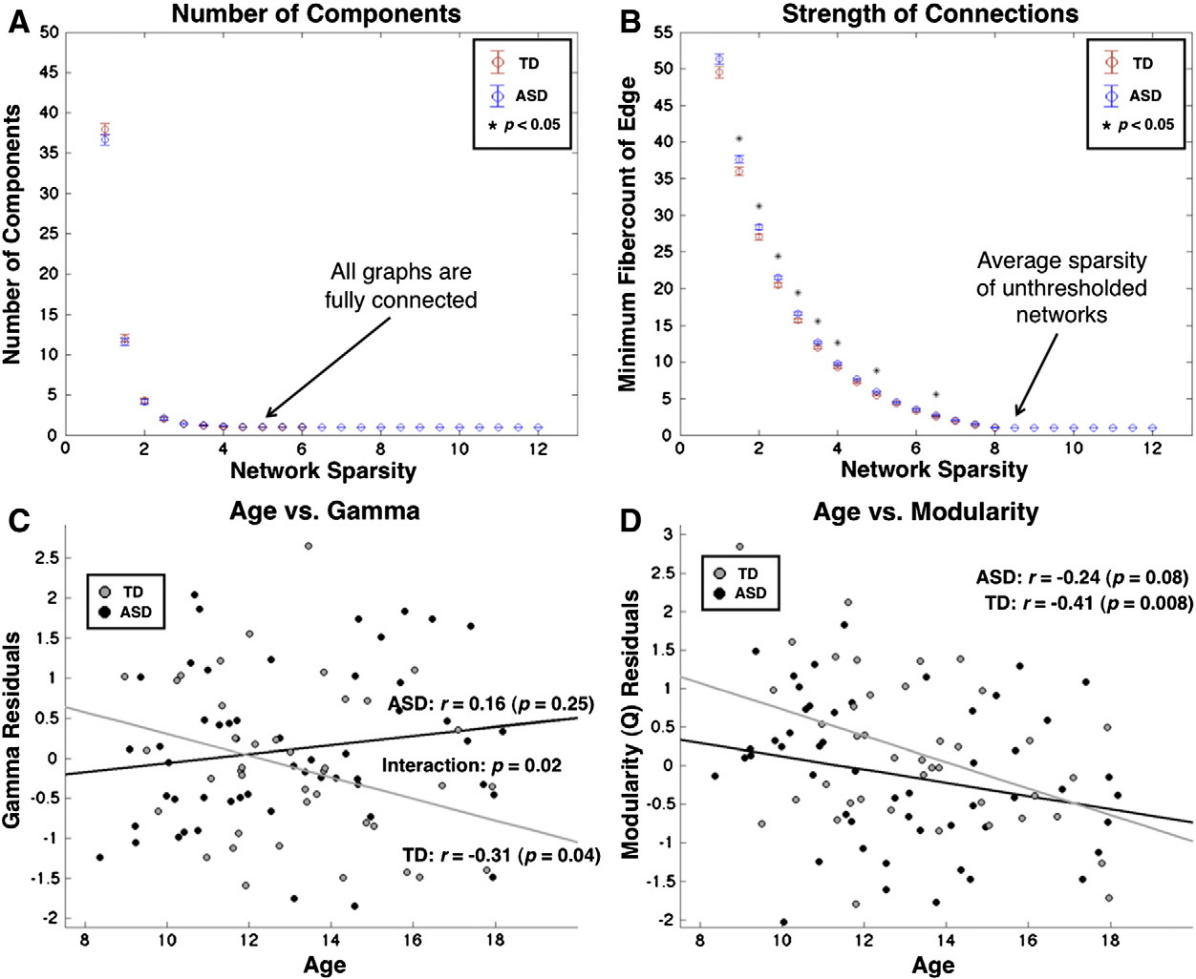
Graph theoretical metrics of structural networks. (A) Average and standard error for TD (red) and ASD (blue) number of components, (B) minimum fiber count for edges, as a function of network sparsity. Number of components and minimum correlation strength are shown between 1% and 12% network sparsity in 0.5% increments. Significant between group differences (*p* < 0.05) are indicated by *. Gamma (C) and modularity (D) residuals after regressing out mean and relative values are displayed as a function of age in the TD (gray) and ASD (black) groups.

For structural networks, we examined the same six global network properties as functional networks (CC, CPL, lambda, gamma, small worldness and modularity Q values) averaged between 5% and 8.5% sparsity in 0.5% increments. Structural networks were then binarized in order to maintain maximum comparability to equivalent functional networks. A sparsity level of 5% represented the minimum sparsity level at which every subject's graph was fully connected (Fig. 6A) and 8.5% represented the average unthresholded sparsity of all subject's structural matrices (Fig. 6B). Two sample *t*-tests were performed on these six metrics between subjects for averaged metrics (with FDR correction) as well as at each sparsity level.

### 2.9 Correlation between fiber count and functional connectivity strengths

**Fig. 7:**
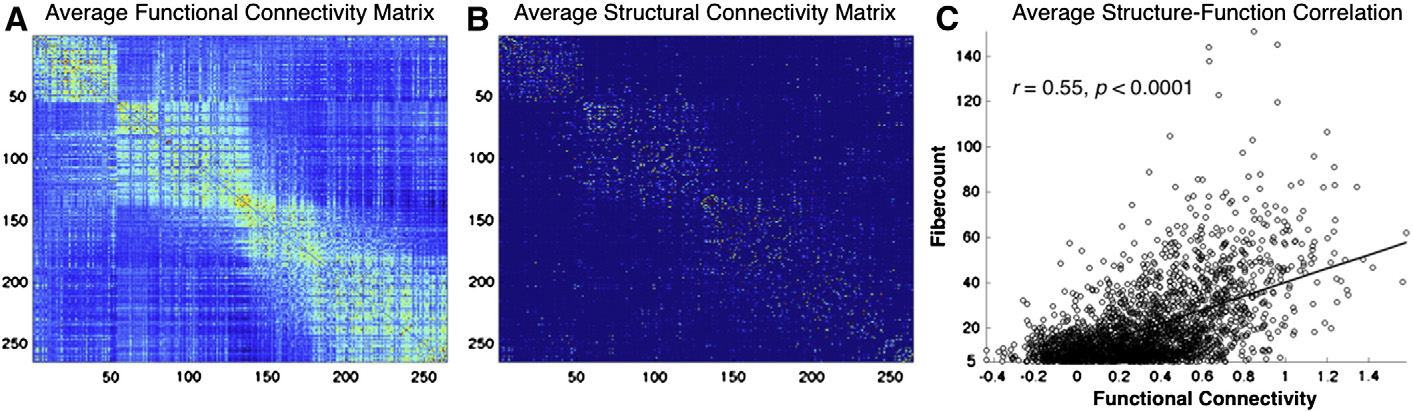
Structure–function correlations. (A) Average functional connectivity and (B) structural fiber connectivity matrices after reorganizing by modular organization for functional networks. Correlation between structure and function for group average connections with a minimum average of 5 fibers.

Fiber counts of every connection with an average of at least 5 fibers were correlated with functional connectivity strengths for each of the 35 ASD and 35 TD subjects (Fig. 7). Additionally, fiber count/functional connectivity correlations were computed for within- and between-module connections and specifically for within-module connections with lower levels of functional connectivity as identified in Fig. 1C. These structure–function correlations were z transformed, then compared between groups (with two-sample *t*-tests).

### 2.10 Principal component analysis of functional and structural network properties

**Fig. 8:**
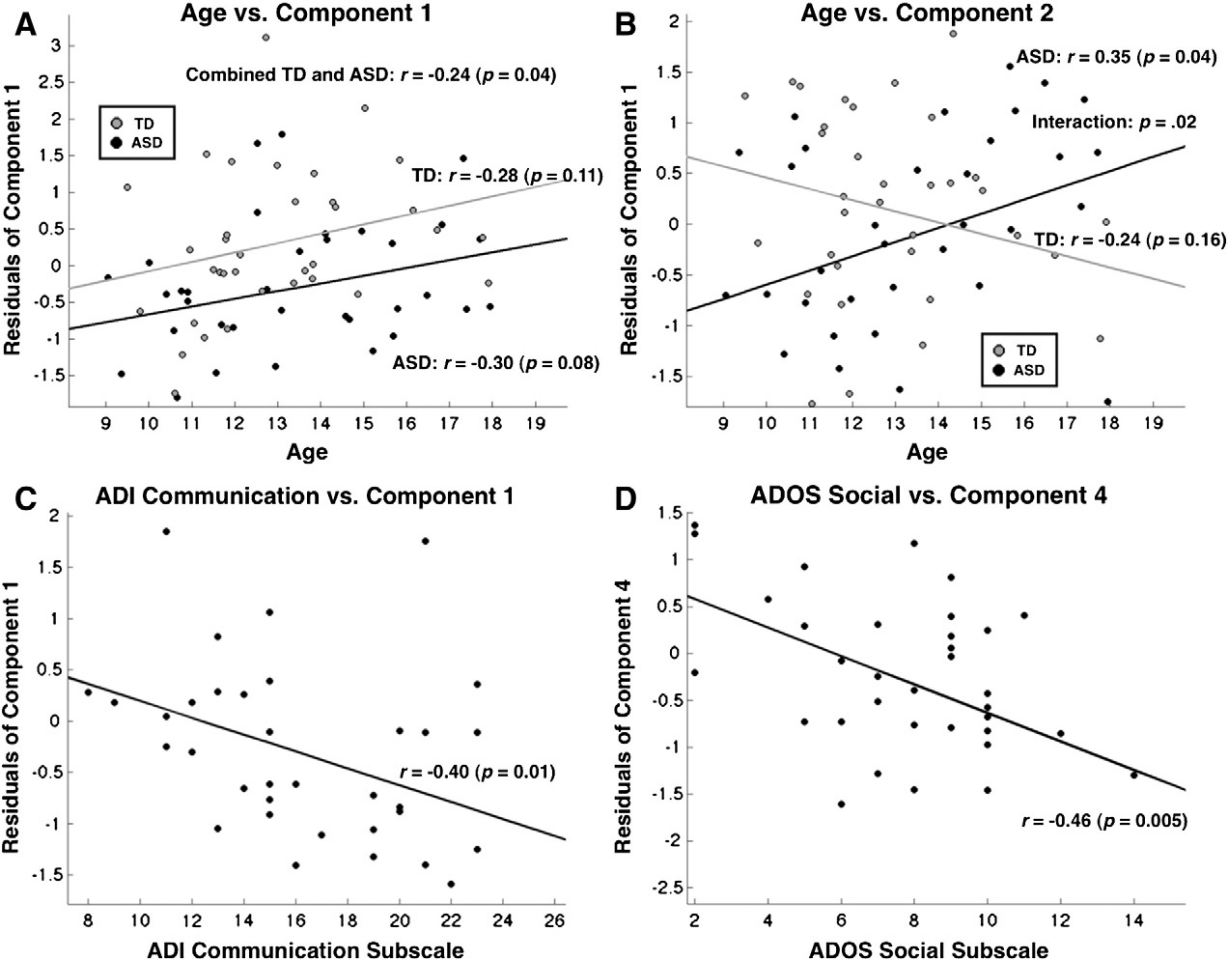
Relationships between principal components of structural and functional network properties, age and ASD symptom severity. (A) Component 1 and (B) Component 2 residuals after regressing out mean motion are displayed as a function of age in the TD (gray) and ASD (black) groups. (C) Residuals of component 1 after regressing out mean motion and age are displayed as function of the Autism Diagnostic Interview (ADI) communication subscales. (D) Residuals of component 4 after regressing out mean motion and age are displayed as a function of the Autism Diagnostic Observation Scale (ADOS) social subscales.

We ran an exploratory principal component analysis (PCA) on the six average functional global graph metrics and the six average structural graph global metrics across all 70 subjects with PASW Statistics 18, Release Version 18.0.3 (SPSS, Inc., Chicago, IL). Values for the first four of 12 total components were computed for each subject and compared between groups (two sample *t*-tests, with FDR correction), and correlated with chronological age after regressing out mean relative motion (with FDR correction; Table 3; Fig. 8A,B). The two components that significantly differed between groups were also tested for correlation with symptom severity (as measured by the social and communication subscales of the ADOS and ADI; with FDR correction) within the ASD group after regressing out mean relative motion and age (Table 3; Fig. 8C–D).

### 2.11 Graph renderings and visualizations

Renderings were generated from scripts in the UCLA Multimodal Connectivity Package (http://github.com/jbrown81/umcp) and through the UCLA Multimodal Connectivity Database (http://umcd.humanconnectomeproject.org), which use matplotlib (http://matplotlib.sourceforge.net) and networkX (http://networkx.lanl.gov).

### 2.12 Data sharing

All of the connectivity matrices used in this study are freely available for download at the UCLA Multimodal Connectivity Database (Brown et al., 2012; http://umcd.humanconnectomeproject.org).

## 3 Results

### 3.1 Functional connectivity matrices

Over the course of 100 runs of the modularity algorithm on the average functional connectivity matrix, four communities were detected over 90% of the time. The normalized mutual information between the representative partition and 99 additional iterations of the modularity algorithm was 0.73 +/− 0.05 (mean +/− standard deviation; NMI ranges from 0 to 1, with 1 representing a perfect similarity). There was moderate similarity between the representative group average modularity partition and modularity partitions of individual subjects (ASD group NMI = 0.285 +/− 0.07 and TD group NMI = 0.288 +/− 0.08; *p* = 0.86). The average NMI between randomized individual matrices and the representative group average modularity partition was equal 0.04, suggesting that the similarity between individual matrices and the group average was above chance.

The order of nodes in the matrix was reorganized to reflect the community structure of the representative group average modularity partition (Fig. 1A). The four communities corresponded to visual, sensorimotor and default systems as well as a largely frontal system corresponding to the task positive control/attention network (color boxes in Fig. 1A and displayed in 3D brain space in Fig. 1B).

We first examined pairwise differences in the connectivity matrices by directly comparing correlation strengths between groups for each connection (Fig. 1C) and separating differences based on within- and between-community connections. We found that the TD group exhibited 5.4 times as many stronger (*p* < 0.05, uncorrected) within-module positive connections as the ASD group (Fig. 1C,D). This was most pronounced in the default (265 (10.1%) connections stronger for TD group vs. 15 (0.5%) stronger for ASD), visual (107 (7.5%) connections stronger for TD group vs. 7 (0.5%) stronger for ASD) and sensorimotor systems (84 (2.5%) connections stronger for TD group vs. 33 (1.0%) stronger for ASD; Fig. 1D, left). There were a similar number of stronger within-module connections for the attention/control network (34 (2.3%) connections stronger for TD group vs. 41 (2.3%) stronger for ASD). Additionally, the TD group exhibited 4.4 times as many stronger (*p* < 0.05, uncorrected) negative (i.e., weaker) between-module connections. This was most prominent for connections between other systems and the default (670 (4.8%) for TD > ASD vs. 152 (1.1%) for ASD > TD) system, but was also true for visual (383 (3.4%) for TD > ASD vs. 135 (1.2%) for ASD > TD), sensorimotor (479 (3.2%) for TD > ASD vs. 154 (1.0%) for ASD > TD), and attention (276 (2.4%) for TD > ASD vs. 165 (1.5%) for ASD > TD) systems (Fig. 1D, right). Thus, there was a pattern of weaker within-network positive connectivity and weaker between-network negative connectivity for children and adolescents with ASD.

We sorted within and between-module differences as a function of average correlation strengths (Fig. 2A). Connections where the TD group had stronger positive within-module connectivity tended to have higher average correlation strengths than connections where the ASD group had stronger within-module connectivity (TD = 0.26 +/− 0.19, ASD = 0.16 +/− 0.17, *p* = 0.0002). Between-module connections where the TD group had stronger negative connectivity were more negative than the connections where the ASD group had stronger negative connections (TD = − 0.16 +/− 0.09, ASD = − 0.08 +/− 0.06, *p* < 0.0001). We found no significant differences (all *p* > 0.25) for the average Euclidean distance of connections that differed between groups for stronger positive within-module connectivity or stronger negative between-module connectivity (Fig. 2B).

### 3.2 Functional connectivity graph metrics

There were group differences in nearly all graph theoretical metrics for functional networks over a range of network sparsities (Fig. 3) and averaged across sparsity levels (Table 2). Clustering coefficient was significantly lower (FDR *q* < 0.05) in the ASD group (Fig. 3C) and although lambda was lower in the ASD group at higher sparsity levels (Fig. 3D), there was only a trend for lower average gamma. Both CPL and gamma were lower in the ASD group over the entire range of sparsities (FDR *q* < 0.05; Fig. 3E,F) and averaged across sparsity levels (Table 2). Both TD and ASD subjects had functional networks in the small world range (the ratio of lambda to gamma being greater than 1.2). However, small worldness was not significantly different between groups. Modularity (Q values) was significantly lower (FDR *q* < 0.05) in the ASD group at every sparsity level and averaged across sparsity levels (Fig. 3H). In addition to averaging metrics across this sparsity range, metrics were also integrated across sparsity levels as in Ginestet et al. (2011), and then compared between groups. There was a 0.99 correlation between averaged and integrated metrics. Additionally, there were no alterations in any of the results reported above when using integrated metrics.

Given the significant between-group differences in global metrics for CC, CPL and modularity, we sought to determine whether specific nodes contributed to these global differences. Therefore, we compared nodal measures of local interconnectivity, hubness, and connection diversity by calculating each node's clustering coefficient, betweenness centrality, and participation coefficient (Guimerà et al., 2005) between groups averaged over the same range of thresholds (Fig. 4). We report the number of nodes with significant between-group differences (*p* < 0.05 uncorrected and FDR corrected; *q* < 0.05, *p* < 0.0013). ASD subjects had lower nodal CC in 21 visual (4 FDR: right occipital fusiform gyrus and left and right inferior lateral occipital cortex), 20 default (3 FDR: medial prefrontal cortex, ventromedial prefrontal cortex and left frontal orbital cortex), and 10 sensorimotor nodes (1 FDR: left superior parietal lobule; Fig. 4A). Participation coefficients were higher for the ASD group in 26 default (3 FDR: medial prefrontal cortex and left frontal orbital cortex), 10 sensorimotor (3 FDR: left postcentral gyrus, left superior parietal lobule and brainstem) and 9 attention (0 FDR) nodes (Fig. 4B). There were no differences in nodal betweenness centrality that survived FDR correction.

### 3.3 Structural connectivity matrices

The Louvain modularity algorithm detected between 8 and 10 communities for the average fiber connectivity matrix over 100 runs. Nine communities were detected in over 80% of the runs and these communities corresponded to sets of lateralized nearby brain regions (Fig. 5B). The average fiber structural connectivity matrix for all TD and ASD subjects is shown in Fig. 5A, after reordering the nodes by the community structure of a representative modularity partition.

The calculated normalized mutual information between the representative structural modularity partition and 99 additional iterations of the modularity algorithm was 0.84 +/− 0.04. There was also high similarity between the representative group average modularity partition and modularity partitions of individual subjects (ASD group NMI = 0.66 +/− 0.05; TD group NMI = 0.68 +/− 0.05; *p* = 0.14).

We first examined the connectivity matrices by directly comparing the number of fibers, average FA, and average MD values for each connection between groups after masking for regions that contained an average of at least 5 fibers (corresponding to 5.75% of all possible connections). We found that the ASD group had 4.2 times as many connections with significantly (*p* < 0.05, uncorrected) more fibers than the TD group (106 ASD > TD vs. 25 TD > ASD; Fig. 5C). We also found that the ASD group had 1.6 times as many connections with lower FA (67 TD > ASD vs. 41 ASD > TD) and 6.2 times as many connections with higher MD (112 ASD > TD vs. 18 ASD > TD; Fig. 5D).

The average number of fibers or Euclidean distance of the connection did not differ for connections where the ASD group had more fibers compared to connections where the TD group had more fibers (number of fibers: TD > ASD = 30.0 +/− 26.6, ASD > TD = 26.3 +/− 25.9, *p* = 0.53; Euclidean distance: TD > ASD = 25.9 +/− 12.8, ASD > TD = 34.8 +/− 26.3, *p* = 0.11). Connections where the TD group had higher white matter integrity (lower MD) had a higher average number of fibers than connections where the ASD group had higher white matter integrity (ASD > TD = 32.5 +/− 28.8, TD > ASD = 11.8 +/− 6.0, *p* = 0.003), but did not differ based on Euclidean distance (ASD > TD = 30.3 +/− 21.5, TD > ASD = 35.2 +/− 7.5, *p* = 0.34).

### 3.4 Structural connectivity graph metrics

Although gamma (normalized characteristic path length) was similar for structural and functional networks (~ 1.2 for structural vs. ~ 1.1 for functional), lambda (normalized clustering coefficient) was much higher in structural networks (~ 5.4 for structural vs. ~ 2.2 for functional). Therefore, structural networks displayed higher levels of small worldness compared to functional networks in both TD and ASD groups. Measures for average structural CC, gamma, CPL, lambda and small worldness did not significantly differ between groups (Table 2). Modularity Q values were higher in the TD group on average, but this did not survive FDR correction.

Given the previous reports of modularity decreasing with age and global efficiency increasing with age in structural networks (Hagmann et al., 2010), we ran post-hoc analyses correlating these metrics with chronological age in each group. Higher modularity in the TD group was actually driven by the younger TD participants, whereby, controlling for motion, modularity was significantly negatively correlated with age in the TD group (*r* = − 0.41, *p* = 0.008; Fig. 6D) yet was only trending toward a negative correlation with age in the ASD group (*r* = − 0.24, *p* = 0.08) although the interaction was not significant (*p* = 0.37). Similarly, there were no group differences for CPL or lambda, but age was negatively correlated with CPL and lambda in the TD group (controlling for motion: CPL: *r* = − 0.34, *p* = 0.03; lambda *r* = − 0.31, *p* = 0.04), and CPL and lambda were positively correlated with age in the ASD group (controlling for motion: CPL: *r* = 0.22, *p* = 0.12; gamma: *r* = 0.16, *p* = 0.25; Fig. 6C) whereby there was a significant group by age interaction for CPL (*p* = 0.01) and gamma (*p* = 0.02).

### 3.5 Structure–function correlation

When comparing correlations between fiber counts and functional connectivity strength between groups, we found that both groups exhibited moderate, yet highly significant (all subjects *p* < 0.001), levels of structural–functional connectivity correlations (TD: *r* = 0.32 +/− 0.03, ASD: *r* = 0.32 +/− 0.04, *p* = 0.77 for the group difference). Furthermore, there were no group differences when structure–function correlations were assessed for both within- and between-functional module connections (within-module: TD: *r* = 0.28 +/− 0.04, ASD: *r* = 0.28 +/− 0.05, *p* = 0.98 for the group difference and between-module TD: *r* = 0.28 +/− 0.05, ASD: *r* = 0.26 +/− 0.05, *p* = 0.32 for the group difference) or specifically for within-module connections exhibiting lower levels of functional connectivity (TD: *r* = 0.30 +/− 0.12, ASD: *r* = 0.31 +/− 0.11, *p* = 0.77 for the group difference).

### 3.6 Principal component analysis of structural and functional metrics

To identify key factors underlying correlated graph metrics and to better understand relationships between structural and functional network properties, we entered the six functional and six structural average global graph metrics for all 70 subjects into an exploratory principal component analysis. We only examined the first four components, as they explained the vast majority (88%) of the variance in the data (Table 3). The first component (accounting for 33.9% of the variance) broadly weighted functional metrics positively and structural metrics negatively. This component was significantly lower in the ASD group (covarying for mean head motion, *p* = 0.009, FDR *q* < 0.05) and negatively related to symptom severity, as measured by the ADI social subscale (covarying for age and mean head motion, *r* = − 0.4, *p* = 0.01; FDR *q* < 0.05; Fig. 8C). The first component was also positively correlated with age in both groups (covarying for mean motion; all: *r* = 0.24, *p* = 0.04, TD: *r* = 0.28, *p* = 0.11 and ASD: *r* = 0.30, *p* = 0.08; Fig. 8A). The second component weighted all functional and structural metrics positively, and although it did not differ between groups, there was a significant interaction with age (covarying for mean head motion; *p* = 0.02), whereby the second component was significantly positively correlated with age in the ASD group (*r* = 0.35, *p* = 0.04) and slightly negatively correlated with age in the TD group (*r* = − 0.24, *p* = 0.16). The third component, positively weighting functional CC/modularity and negatively weighting functional CPL, did not differ between groups. The fourth component, positively weighting structural modularity and negatively weighting structural CPL, was significantly lower in the ASD group (*p* = 0.007; FDR *q* < 0.05) and was negatively correlated with symptom severity as measured by the ADOS social and communication subscales (covarying for age and mean head motion, ADOS social: *r* = − 0.46, *p* = 0.005; FDR *q* < 0.05, Fig. 8D).

## 4 Discussion

Previous neuroimaging studies on ASD have reported reduced functional and structural connectivity both within and between specialized brain systems (Vissers et al., 2012), suggesting ASD is a network disorder (Müller, 2007). Here we expand upon previous findings of lower functional and structural connectivity in ASD by characterizing higher-level network properties using tools derived from the physics of complex networks (Rubinov and Sporns, 2009). We report alterations in community organization of functional networks, as well as in the balance of local and global efficiency within and between structural and functional networks in children and adolescents with ASD relative to their typically-developing counterparts.

### 4.1 Functional connectivity alterations

We detected robust reductions in positive functional connectivity within major functional systems (i.e., functional integration) in individuals with ASD. Reduced functional connectivity was most prominent in the default system, consistent with multiple studies that have found reduced DMN connectivity in ASD (Kennedy and Courchesne, 2008; Assaf et al., 2010; Wang et al., 2010). However, we also found weaker connectivity within visual (largely secondary areas) and sensorimotor systems, supporting more widespread alterations in functional connectivity as found by Villalobos et al. (2005), Mostofsky et al. (2009), and Anderson et al. (2011b). Relatively few alterations were observed in the frontal attention/cognitive control network, which might reflect relatively intact cognitive skills in high-functioning individuals with ASD (Kennedy and Courchesne, 2008).

Interestingly, individuals with ASD also show reduced negative (i.e., more positive) connectivity *between* systems. Consistent with previous findings in the task positive and default mode networks in ASD (Rudie et al., 2012a), weaker negative connectivity between communities suggests that specific functional systems are less distinct or functionally segregated from one another. Although there is some controversy regarding the proper interpretation of negatively correlated brain regions when using global signal regression (GSR; Murphy et al., 2009; Fox et al., 2009), anticorrelations are detected without GSR (Chang and Glover, 2009; Anderson et al., 2011c; Smith et al., 2012) and GSR maximizes the specificity of positive resting-state correlations in real and simulated data (Fox et al., 2009; Weissenbacher et al., 2009). Interestingly, reduced negative connectivity was recently shown to be useful for diagnostic classification of autism in analyses without GSR (Anderson et al., 2011b). Therefore, although it is unclear whether widespread differences in negatively connected regions are exaggerated by GSR, differences in negative connectivity between distinct functional systems are likely important for understanding ASD neurobiology.

Although most previous functional connectivity studies of ASD have reported underconnectivity of long-range (i.e., anterior–posterior or interhemispheric) connections, it has also been widely hypothesized that ASD may be related to *overconnectivity* of short-range connections (Belmonte et al., 2004; Courchesne and Pierce, 2005; Geschwind and Levitt, 2007). Previous neuroimaging studies have found increased short-range connections in neurotypical children versus adults (Fair et al., 2009; Supekar et al., 2009) but findings are somewhat mixed in individuals with ASD (Paakki et al., 2010; Shukla et al., 2010). Consistent with a recent study (Anderson et al., 2011b) we found that even short-range functional connections are reduced in ASD. Of course, this does not exclude the possibility that local connections at the neuronal or minicolumnar level could be enhanced in ASD (Casanova et al., 2002).

In examining graph metrics of functional networks, we found that individuals with ASD had lower clustering (i.e., local efficiency), especially in nodes within the default systems and secondary visual areas. Individuals with ASD displayed a less robust modular organization (i.e., communities were less distinct) and there was a tendency for nodes in the default and sensorimotor systems to interact more with other communities as measured by higher nodal participation coefficients. Finally, we found that functional brain networks in individuals with autism had shorter average path lengths (i.e., higher levels of global efficiency) as well as normalized characteristic path lengths. Randomly connected networks tend to have short path lengths (Sporns, 2011) suggesting the possibility that higher global efficiency in functional networks may simply reflect a less organized or more random distribution of functional edges. This is consistent with a study finding decreased complexity or increased randomness in resting-state fMRI timeseries of individuals with ASD (Lai et al., 2010).

Previous functional graph theory studies in typical development (Fair et al., 2009; Supekar et al., 2009) did not find differences in local or global efficiency between children and adults. However, in addition to decreases in long-range connectivity, these developmental studies reported increased local connectivity, which may explain these null findings. Additionally, the extent to which these previously reported developmental differences are attributable to motion artifacts is unclear given that subtle motion spikes tend to reduce long range connectivity yet *increase* local connectivity (Power et al., 2012). Given our careful consideration of head motion through regression of motion spikes and covarying for motion at the group level, as well as the fact that we found both reduced long- and short-range connectivity in ASD, it is unlikely that our findings are related to between-group differences in motion. Although future work is needed to further examine developmental changes in the context of more stringent motion correction, our findings in ASD are somewhat consistent with the studies in typical development reporting reduced integration and segregation of functional systems in children relative to adults. Therefore, although functional networks in ASD may be ‘immature’ in some ways (i.e., reflect an earlier developmental stage as far as reduced integration/segregation of major systems), they may also be fundamentally different from neurotypical individuals from a network perspective (i.e., reduced local efficiency yet increased global efficiency).

### 4.2 Structural connectivity alterations

For structural connectivity measures derived from diffusion MRI, we found reduced integrity in short- and long-range white matter tracts in ASD in line with previous studies (e.g., Barnea-Goraly, 2003; Shukla et al., 2010). We found more robust differences in MD than FA, which has been reported in several previous DTI studies (Sundaram et al., 2008; Groen et al., 2011). Interestingly, despite the fact that white matter integrity was generally reduced, we found evidence for increased fiber counts in ASD, which may relate to early reports of increased regional white matter (Herbert et al., 2004) and more recent reports of increased fiber counts in certain tracts in ASD (Pugliese et al., 2009). Although white matter integrity is lower in children compared to adults, fiber counts increase during development (Lebel et al., 2012). Therefore, like functional networks, some alterations in ASD may reflect immaturity, while other alterations are likely to reflect *aberrant* processes.

Structural networks displayed high levels of local and global efficiency in both the TD and ASD groups. Given previous reports of decreasing modularity and increasing global efficiency of structural networks with development (Hagmann et al., 2010), we examined the relationship between age and modularity/global efficiency in each group. We found that in the TD group, modularity sharply decreased with age whereas global efficiency increased with age, consistent with previous reports. In the ASD group, modularity decreased at a slower rate and, contrary to findings in the TD group, global efficiency actually decreased with age. It should be noted that global efficiency in structural networks likely reflects a different underlying substrate than global efficiency in functional networks given the physical wiring costs of structural networks (Bassett et al., 2010; Fornito et al., 2011). Thus, despite similar levels of local and global efficiency in structural networks across both groups, it appears as though network efficiency does not appropriately shift from a more local to a more distributed pattern during development in individuals with ASD (Hagmann et al., 2010).

One potential limitation of our analyses is that we chose to use a set of spherical, functionally-based ROIs instead of more traditional block or atlas based regions, which would have allowed for the inclusion of deeper white matter and, relatedly, increased signal to noise in the tractography analyses. This decision was made in order to allow for a more direct comparison between structural and functional connectivity. Additionally, previous structural connectivity studies have used fibers that terminate at this gray–white boundary because they are the most reliable/likely estimates of cortico-cortical connectivity (Hagmann et al., 2008; Honey et al., 2009). However, future work should incorporate other parcellation schemes that include deeper white matter while also allowing for a direct comparison of structure and function.

### 4.3 Relationships between structure and function

When relating structural and functional connectivity, we found that measures of fiber counts and functional connectivity strength were moderately positively correlated in both groups with no group differences regardless of whether the connections were within or between modules or whether we only included connections with lower levels of functional connectivity. This finding, in addition to the fact that we generally saw higher fiber counts in ASD, suggests that alterations in functional connectivity in ASD are not directly related to alterations in fiber organization.

In order to relate structural and functional network properties, we performed a principal component analysis. Interestingly, we found that the largest underlying factor *inversely* weighted structural and functional network properties. This component inversely weighted local and global efficiency (i.e., positively weighted both CC and CPL) within functional and structural networks and was positively correlated with age. Although preliminary, this finding suggests that structural networks become more globally efficient, yet less locally efficient, during development while functional networks display a relative inverse pattern. This component was reduced in ASD and inversely related to social and communicative behavior, suggesting that the balance between structural and functional network properties is related to social impairments in ASD. Further highlighting differential age-related trajectories for functional and structural network properties, the second component, which positively weighted both structural and functional metrics, decreased with age in the TD group while it increased with age in the ASD group. Interestingly, a previous study of multiple sclerosis (Hawellek et al., 2011) found that disruption of white matter pathways actually leads to increased functional connectivity in multiple networks including the DMN, which further highlights a divergence between the structural and functional connectomes. However, it should be noted that here we found inherently different sparsity ranges for structural and functional networks. Direct comparison of structural to functional graphs at different sparsities is problematic given that graph theoretical metrics can vary as a function of network sparsity. Our PCA method attempts to overcome the confound of direct comparison between differentially sparse structural and functional graphs in order to integrate information across modalities.

Finally, the fourth component, which positively weighted local and global efficiency in structural networks, was reduced in ASD and inversely related to social and communicative symptom severity. Therefore, an underlying factor positively influencing both local and global efficiency in structural networks may also relate to disrupted social behavior in ASD.

### 4.4 Future directions

Future studies should characterize younger and/or lower functioning individuals with ASD since our findings are limited to high-functioning children and adolescents with ASD. For example, studies examining infants at high risk for ASD may be useful for developing biomarkers to aid in earlier diagnosis and treatment. Future studies may also benefit from advances in imaging acquisition (Feinberg et al., 2010), more flexible modeling approaches (Smith et al., 2012), and large-scale studies involving collaboration between institutions (Biswal et al., 2010). Additionally, comparisons with other neuropsychiatric disorders, and teasing apart underlying mechanisms such as genetic risk factors (Brown et al., 2011; Dennis et al., 2012; Rudie et al., 2012b) will all be crucial for a more complete characterization of brain network abnormalities in ASD.

### 4.5 Conclusions

To our knowledge, this is the first study to use complex network analyses to examine both structural and functional brain networks in autism. We found significant reductions in local efficiency and modularity within several functional networks. ASD children and adolescents also displayed atypical age-related changes in the balance of local and global efficiency between structural and functional networks. Further, this imbalance was related to the severity of socio-communicative deficits in individuals with ASD. Our findings suggest that complex network modeling of structural and functional brain organization will yield a better understanding of the neural basis of ASD and other neuropsychiatric disorders. Ultimately, a more cohesive framework for understanding brain alterations in ASD may inform the design of more sophisticated diagnostic tools and targeted interventions.

## Figures and Tables

**Table 1:** Mean, standard deviation and range of sample descriptives.

Characteristic	Typically developing	Autism spectrum	*p* value
*Resting state (RS) sample*			
Sample size	37.0	42.0	
Number of females	6.0	6.0	0.81
Age	13.0 +/− 2.0, 9.5–17.8	13.5 +/− 2.4, 9.3–17.9	0.30
Verbal IQ	108.4 +/− 11.0, 86–127	103.6 +/− 12.7, 79–132	0.07
Performance IQ	105.2 +/− 11.9, 76–129	103.5 +/− 14.4, 72–134	0.57
Full scale IQ	106.8 +/− 10.0, 84–128	103.3 +/− 14.0, 79–134	0.19
Mean relative head motion (mm)	0.09 +/− 0.07, 0.03–0.37	0.11 +/− 0.07, 0.04–0.37	0.33
Maximum relative head motion (mm)	0.66 +/− 0.63, 0.10–2.46	0.83 +/− 0.61, 0.15–2.50	0.25
ADOS (Comm + Soc)	N/A	11.1 +/− 3.9, 2.19	N/A
ADI total	N/A	47.9 +/− 9.8, 23–63	N/A

*Diffusion tensor imaging (DTI) sample*			
Sample size	43.0	51.0	
Number of females	7.0	6.0	0.53
Age	13.1 +/− 2.4, 9.0–18.0	13.0 +/− 2.8, 8.4–18.2	0.82
Verbal IQ	108.2 +/− 12.6, 86–131	104.3 +/− 13.9, 83–141	0.15
Performance IQ	105.9 +/− 13.2, 76–134	105.0 +/− 14.6, 72.135	0.74
Full scale IQ	108.2 +/− 12.5, 84–134	104.1 +/− 13.2, 79–132	0.15
Mean relative head motion (mm)	0.41 +/− 0.13, 0.27–0.81	0.42 +/− 0.11, 0.26–0.66	0.71
Maximum relative head motion (mm)	1.52 +/− 0.69, 0.96–4.62	1.71 +/− 0.81, 0.90–4.86	0.23
ADOS (Comm + Soc)	N/A	10.8 +/− 3.6, 2–19	N/A
ADI total	N/A	47.4 +/− 11.6, 16.68	N/A

*DTI and RS sample*			
Sample size	35.0	35.0	
Number of females	6.0	5.0	0.74
Age	13.0 +/− 2.1, 9.5–18.0	13.4 +/− 2.6, 9.1–18.2	0.51
Verbal IQ	108.3 +/− 11.5, 86–127	102.9 +/− 13.9, 79–132	0.08
Performance IQ	105.4 +/− 12.1, 76–129	105.1 +/− 14.5, 72–135	0.94
Full scale IQ	107.7 +/− 11.0, 84–128	103.2 +/− 13.6, 79–132	0.13
RS mean relative head motion (mm)	0.09 +/− 0.07, 0.03–0.37	0.10 +/− 0.06, 0.04–0.29	0.38
RS maximum relative head motion (mm)	0.67 +/− 0.65, 0.10–2.46	0.80 +/− 0.57, 0.15–2.10	0.37
DTI mean relative head motion (mm)	0.39 +/− 0.12, 0.27–0.78	0.40 +/− 0.09, 0.26–0.59	0.81
DTI maximum relative head motion (mm)	1.45 +/− 0.59, 1.00–4.62	1.44 +/− 0.47, 0.90–3.17	0.99
ADOS (Comm + Soc)	N/A	11.3 +/− 3.8, 2–19	N/A
ADI total	N/A	47.8 +/− 9.8, 23–61	N/A

Data is mean +/− standard deviation, minimum–maximum. Columns on the right display *p*-values for two sample *t*-tests for each sample characteristic except for sex, which displays *p*-values from a Chi square test.

**Table 2:** Mean and standard deviation of functional and structural graph metrics.

Characteristic	Typically developing	Autism spectrum	*p* value
*Functional (42 ASD vs 37 TD)*			
Clustering coefficient	0.56 +/− 0.03	0.54 +/− 0.03	0.012⁎
Characteristic path length	1.92 +/− 0.05	1.89 +/− 0.05	0.02⁎
Lambda	2.18 +/− 0.12	2.13 +/− 0.13	0.070
Gamma	1.09 +/− 0.03	1.07 +/− 0.03	0.02⁎
Small worldness	2.00 +/− 0.10	1.98 +/− 0.12	0.420
Modularity (Q)	0.40 +/− 0.03	0.38 +/− 0.03	0.008⁎

*Structural (51 ASD vs 43 TD)*			
Clustering coefficient	0.46 +/− 0.01	0.46 +/− 0.01	0.750
Characteristic path length	2.77 +/− 0.04	2.77 +/− 0.04	0.490
Lambda	5.44 +/− 0.23	5.39 +/− 0.19	0.270
Gamma	1.24 +/− 0.02	1.24 +/− 0.02	0.990
Small worldness	4.38 +/− 0.16	4.33 +/− 0.13	0.120
Modularity (Q)	0.68 +/− 0.01	0.67 +/− 0.01	0.030

Data is mean +/− standard deviation. *p* values were generated from two-sample *t*-tests performed on each metric averaged over a range of sparsity thresholds.

⁎ Survives FDR (*q* < 0.05).

**Table 3:** Principal component analysis of functional and structural network metrics.

	Component 1	Component 2	Component 3	Component 4
Functional CC	**0.863**	**0.369**	**− 0.33**	− 0.008
Functional CPL	**0.81**	**0.317**	**− 0.486**	− 0.012
Functional lambda	**0.551**	**0.471**	**0.672**	0.047
Functional gamma	**0.813**	**0.328**	**− 0.471**	− 0.013
Functional small worldness	0.174	**0.327**	**0.92**	0.05
Functional modularity (Q)	**0.783**	**0.418**	**0.363**	− 0.051
Structural CC	**− 0.44**	**0.415**	− 0.221	0.04
Structural CPL	**− 0.436**	**0.656**	− 0.051	**− 0.604**
Structural lambda	**− 0.441**	**0.775**	− 0.121	**0.329**
Structural gamma	**− 0.455**	**0.671**	− 0.046	**− 0.571**
Structural small worldness	**− 0.337**	**0.648**	− 0.122	**0.593**
Structural modularity (Q)	**− 0.406**	**0.373**	− 0.029	**0.314**
Total variance explained	33.90%	25.50%	17.40%	10.50%
Relationship with diagnosis	***b* =** −**0.30, *p* = 0.009**⁎	*b* = − 0.13, *p* = 0.30	*b* = 0.01, *p* = 0.95	***b* =** −**0.32, *p* = 0.007**⁎
Correlation with age (All)	*r* = 0.24, *p* = 0.04	*r* = 0.07, *p* = 0.34	*r* = − 0.02, *p* = 0.87	*r* = − 0.06, *p* = 0.62
Correlation with age (TD)	*r* = 0.28, *p* = 0.11	*r* = − 0.24, *p* = 0.16	*r* = 0.09, *p* = 0.60	*r* = 0.00, *p* = 1.0
Correlation with age (ASD)	*r* = 0.30, *p* = 0.08	*r* = 0.35, *p* = 0.04	*r* = − 0.11, *p* = 0.53	*r* = − 0.09, *p* = 0.62
Correlation with ADOS social (ASD)	*r* = − 0.04, *p* = 0.81	–	–	*r* = − 0.36, *p* = 0.04
Correlation with ADOS comm (ASD)	*r* = − 0.06, *p* = 0.73	–	–	***r* =** −**0.46, *p* = 0.005**⁎
Correlation with ADI social (ASD)	***r* =** −**0.40, *p* = 0.01**⁎	–	–	*r* = − 0.18, *p* = 0.30
Correlation with ADI comm (ASD)	*r* = − 0.30, *p* = 0.08	–	–	*r* = − 0.11, *p* = 0.53

Top of table displays weighting (bold indicates significant (*p* < 0.05) weight) of structural metrics (clustering (CC), characteristic path lenghts (CPL), lambda, gamma, small worldness and modularity (Q)) on each of the four principal components. Bottom of table shows regression coefficients and *p* values with diagnosis and Pearson correlation values with age (controlling for motion), Autism Diagnostic Interview (ADI) and Autism Diagnostic Observation Scale (ADOS) social and communication subscales with each of the principle components (controlling for age and motion).

⁎ Survives FDR (*q* < 0.05).
